# Heavy Ion Radiation Exposure Triggered Higher Intestinal Tumor Frequency and Greater β-Catenin Activation than γ Radiation in APC^Min/+^ Mice

**DOI:** 10.1371/journal.pone.0059295

**Published:** 2013-03-21

**Authors:** Kamal Datta, Shubhankar Suman, Bhaskar V. S. Kallakury, Albert J. Fornace

**Affiliations:** 1 Department of Biochemistry and Molecular & Cell Biology and Lombardi Comprehensives Cancer Center, Georgetown University, Washington, DC, United States of America; 2 Department of Pathology, Georgetown University Medical Center, Washington, DC, United States of America; 3 Center of Excellence In Genomic Medicine Research, King Abdulaziz University, Jeddah, Saudi Arabia; National Taiwan University, Taiwan

## Abstract

Risk of colorectal cancer (CRC) after exposure to low linear energy transfer (low-LET) radiation such as γ-ray is highlighted by the studies in atom bomb survivors. On the contrary, CRC risk prediction after exposure to high-LET cosmic heavy ion radiation exposure is hindered due to scarcity of *in vivo* data. Therefore, intestinal tumor frequency, size, cluster, and grade were studied in APC^Min/+^ mice (n = 20 per group; 6 to 8 wks old; female) 100 to 110 days after exposure to 1.6 or 4 Gy of heavy ion^ 56^Fe radiation (energy: 1000 MeV/nucleon) and results were compared to γ radiation doses of 2 or 5 Gy, which are equitoxic to 1.6 and 4 Gy ^56^Fe respectively. Due to relevance of lower doses to radiotherapy treatment fractions and space exploration, we followed 2 Gy γ and equitoxic 1.6 Gy ^56^Fe for comparative analysis of intestinal epithelial cell (IEC) proliferation, differentiation, and β-catenin signaling pathway alterations between the two radiation types using immunoblot, and immunohistochemistry. Relative to controls and γ-ray, intestinal tumor frequency and grade was significantly higher after ^56^Fe radiation. Additionally, tumor incidence per unit of radiation (per cGy) was also higher after ^56^Fe radiation relative to γ radiation. Staining for phospho-histone H3, indicative of IEC proliferation, was more and alcian blue staining, indicative of IEC differentiation, was less in ^56^Fe than γ irradiated samples. Activation of β-catenin was more in ^56^Fe-irradiated tumor-free and tumor-bearing areas of the intestinal tissues. When considered along with higher levels of cyclin D1, we infer that relative to γ radiation exposure to ^56^Fe radiation induced markedly reduced differentiation, and increased proliferative index in IEC resulting in increased intestinal tumors of larger size and grade due to preferentially greater activation of β-catenin and its downstream effectors.

## Introduction

Risk of colorectal cancer (CRC) increases after exposure to ionizing radiation (IR) and epidemiological studies in atom bomb survivors and in nuclear workers have shown increased incidence of CRC relative to corresponding non-exposed groups [1]–[4]. However, such radiation exposures were of low linear energy transfer (low-LET) radiation such as γ-ray, which is sparsely ionizing. In outer space galactic cosmic radiation (GCR), unlike solar particle events (SPE), is always present and highly energetic charged (HZE) heavy ions such as ^56^Fe, ^28^Si, and ^16^O contributes significantly to its dose equivalent [5]. While radiation dose during SPE could reach up to 2 Gy behind shielding, long duration space missions are also expected to expose astronauts to considerable levels of cumulative heavy ion radiation doses and are major health concerns [6], [7]. It is estimated that during a 3-year Mars mission ∼30% of an astronaut’s cells will be traversed by HZE nuclei [8], [9]. HZE particles, while passing through shielding materials and tissues, results in not only higher number of ionizing events in close proximity of the primary track but they also generate more secondary delta-ray events compared to γ radiation [10]–[12]. As a result HZE radiation is high-LET and is densely ionizing posing relative to low-LET radiation greater health risk to astronauts. In contrast to our understanding of risks from low-LET radiation, we are hampered by considerable uncertainties regarding risks of CRC from HZE radiation thus limiting astronauts’ stay in space [12].

Uncertainties associated with the prediction of CRC risk from HZE radiation stem from non- availability of *in vivo* mechanistic data in human or in animal models. Because of the limitations in obtaining human data for heavy ion radiation exposure, studies in animal models relevant to molecular pathways involved in colorectal carcinogenesis would aid in generating *in vivo* data for risk estimates. In APC^Min/+^ mice, an important animal model of CRC, intestinal tumors develop in intestinal epithelial cells (IEC) due to dysregulation of IEC proliferation and differentiation. Crypts and villi constitute functional units of intestinal epithelium and are highly dynamic with continuous renewal process led by intestinal stem cells. Proliferative intestinal stem cells, 4 to 16 in number, reside at the bottom of the crypts and gives rise to terminally differentiated enterocytes, goblet cells, enteroendocrine cells, and Paneth cells [13], [14]. The Wnt signaling pathway plays critical roles in maintaining cellular homeostasis of rapidly cycling IEC. Activation of β-catenin, a Wnt signaling component, due either to loss of the adenomatous polyposis coli (APC) gene or to stabilization mutations in β-catenin itself results in loss of normal rhythm of crypt-villi dynamics, uncontrolled IEC proliferation, and aberrant crypt foci (ACF) formation described as precursors of intestinal adenoma and adenocarcinoma in animal models and human [15]–[17]. Loss-of-function mutations in the APC gene have been implicated in familial as well as in sporadic CRC in humans. The APC gene encodes a 312 KDa protein, which modulates the Wnt-signaling pathway via negative regulation of β-catenin-mediated transcriptional activities. Interaction of APC in a complex of axin, glycogen synthase kinase 3β (GSK3β), and β-catenin leads to the phosphorylation and proteasomal degradation of β-catenin. Βeta-catenin, upon loss of APC function, not only escapes proteosomal degradation but it also translocates to the nucleus, interacts with TCF4 and transcribes its target proliferative genes such as c-Myc and cyclin D1 [18]–[20].

The APC^Min/+^ mouse model with a truncation mutation at codon 850 of the APC gene has been extensively used to investigate pathogenesis of CRC and to develop therapeutic and chemopreventive agents for CRC [21], [22]. Unlike in human, the Min (multiple intestinal neoplasia) mice develop adenomas mostly in the small intestine rather than in colon. However, due to similarities with human colorectal carcinogenesis, the APC^Min/+^ (Min) mouse model remains an important tool towards our understanding of colorectal carcinogenesis [23]. The Min mice develop on an average 35 to 45 adenomas and these may appear as early as 60 days after birth. Others and we have reported that exposure of APC^Min/+^ mice to low-LET radiation causes significant increases in intestinal tumors relative to unirradiated controls [24]–[26]. We have also shown that 4 Gy ^56^Fe radiation induced greater intestinal tumorigenesis than equitoxic 5 Gy γ irradiation in these mice [25]. Alteration in molecular pathways leading to CRC initiation and progression has been primarily worked out through *in vitro* and *in vivo* mouse model works. However, our understanding of intestinal tumorigenesis and associated molecular events after exposure to ^56^Fe radiation remains unexplored.

## Materials and Methods

### Animal Breeding and Genotyping

APC^Min/+^ mice were bred and genotyped as described previously [25]. Briefly, male C57BL/6J APC^Min/+^ mice were crossed with the wild-type female C57BL/6J mice and APC^Min/+^ genotyping was performed per Jackson Laboratory (Bar Harbor, ME) protocol. Mice were maintained at the Georgetown University animal facility. Mice were housed in a group of 5 in autoclaved cages and bedding materials in a separate room with 12-h dark and light cycle maintained at 22°C in 50% humidity. All animals were provided certified rodent diet (LabDiet #5053, Brentwood, MO) with filtered water ad libitum and CO_2_ asphyxiation was used for euthanasia. During the post-radiation study period (100 to 110 days) animals were monitored daily for any sign of distress such as hunched posture, ruffled fur, diarrhea, reduced activity, and weight loss (>15%). Any mouse with declining health determined by using the parameters already mentioned was euthanized by CO_2_ asphyxiation and was excluded from the specific study group. Guide for the Care and Use of Laboratory Animals, prepared by the Institute of Laboratory Animal Resources, National Research Council, and U.S. National Academy of Sciences were used as guidelines for our research.

### Irradiation

Female APC^Min/+^ mice (n = 20 mice per group; 6 to 8 week old) were randomly assigned to the study groups and were exposed to whole body γ^2^ or 5 Gy) or ^56^Fe (1.6 or 4 Gy; energy-1000 MeV/nucleon; LET-148 keV/µm) radiation. Control mice were sham-irradiated and all irradiations were performed at 1 Gy/min dose rate as described earlier [27] and a ^137^Cs source was used for γradiation. For ^56^Fe irradiation, mice were shipped to Brookhaven National Laboratory (BNL) animal facility one week prior to radiation exposure and were exposed to ^56^Fe radiation at the NASA Space Radiation Laboratory (NSRL) in BNL and ^56^Fe dosimetry was calculated by the NSRL physics team and is described elsewhere [28]–[30]. Heavy ion ^56^Fe radiation was delivered at the entrance plateau region of the Bragg Peak to achieve a constant LET level [6]. Mice were shipped to and from BNL early in the morning in a temperature-controlled environment along with the respective sham irradiated control groups for same day delivery. Both the GU and the BNL animal care facility are AAALACI (Association for Assessment and Accreditation of Laboratory and Animal Care International) accredited facilities. Ten mice at a time were placed in a pie-shaped clear box with multiple holes and the box with the mice was then placed on a turntable inside a γ-irradiator (^137^Cs) and exposed to 2 or 5 Gy whole body γ irradiation. For ^56^Fe irradiation, each mouse was placed in a small transparent rectangular Lucite box (7.6 cm×3.8 cm×3.8 cm) with multiple holes, and ten of these boxes were packed in a custom-made sample holder made of low-density foam and exposed to 1.6 or 4 Gy ^56^Fe radiation. The sample holder with the mice was then placed in the path of the heavy ion radiation beam (20 cm×20 cm). The ^56^Fe radiation doses were equitoxic to respective γ radiation dose (1.6 Gy ^56^Fe to 2 Gy γ and 4 Gy ^56^Fe to 5 Gy γ radiation) calculated using an RBE factor of 1.25 determined earlier [27].

### Tumor Counts and Sampling

Radiation exposed and age matched sham-irradiated control mice were euthanized by CO_2_ asphyxiation between 100 and 110 days after radiation exposure. The small intestinal tract was surgically removed, divided into duodenum, jejunum, and ilium. These sections were flushed gently with phosphate-buffered saline (PBS) at room temperature. The sections were placed on PBS soaked absorbent paper and cut open longitudinally. Tumors in each section were counted under a dissecting scope (Leica MZ6, Buffalo Grove, IL) by two independent observers blinded to the treatments groups. Data from multiple radiation exposure experiments were pooled to achieve statistical significance. Scale attached to the dissecting scope was used to measure the intestinal tumor size and the number of tumors measuring ≥3 mm in each animal was noted. We also counted tumor clusters, defined as 5 or more tumors without intervening tumor free areas, separately from the tumor size-based counts. Tumors were then carefully dissected under the dissecting scope, flash frozen in liquid nitrogen, and stored at −80°C for immunoblot analysis. Intestinal tissue samples (∼3 cm from jejunum) with tumor and intervening tumor free areas were fixed overnight in 10% buffered formalin. Fixed tissues were embedded in paraffin and 4 µm-thick longitudinal sections were prepared and sections from 2 Gy γ and 1.6 Gy ^56^Fe radiation were used for hematoxylin and eosin (H&E), alcian blue, and immunohistochemistry staining.

### Alcian Blue and H&E Staining

Alcian blue staining of intestinal sections (n = 6 mice per group) having both tumor-free and tumor-bearing areas was performed according to the protocol described earlier [31]. Briefly, tissue sections were deparaffinized, hydrated in distilled water, incubated in 3% glacial acetic acid solution for 3 minute, followed by 30 minute incubation in alcian-blue stain (1% alcian blue in 3% glacial acetic acid; pH 2.5). Sections were counter stained with Nuclear Fast Red (0.1%; Sigma, USA) for 5 min, rinsed in water, dehydrated, and mounted for visualization. H&E staining was performed on all the intestinal sections using standard protocol. A board certified pathologist assessed a total of 34 randomly chosen tumors in each study group in the H&E stained intestinal sections for invasive carcinomas classified by intestinal crypt invasion of muscularis mucosa [32].

### Immunohistochemistry

Unstained sections (n = 6 mice per group) having both tumor-free and tumor-bearing region were used for immunohistochemistry. Briefly, immunostaining for phospho-histone H3 (p-H3, ser-10; Cat# 09-797; dilution: 1∶100; Millipore, Billerica, MA), active-β-catenin (Clone: anti-ABC clone 8E7, Cat#05-665, Millipore, dilution: 1∶100), and cyclin D1 (Clone: EPR2241 (IHC)-32; Cat#04-1151; dilution: 1∶150; Millipore) were performed after antigen retrieval using citrate buffer (pH 6.0; Dako, Carpinteria, CA) in a microwave oven at 100°C for 15 min. After quenching endogenous peroxidase activity and incubation in blocking buffer (0.1% bovine serum albumin in PBS), the sections were exposed to the respective primary antibodies. SuperPicture TM 3^rd^ Gen IHC detection kit (Cat#87-9673; Invitrogen, Carlsbad, CA) was used for signal detection and color development. To determine specificity of the staining, appropriate controls were run in parallel with the experimental sections. Images were captured using bright field microscopy at a magnification of 20X and a total of ten fields of vision (FOV) from the tumor-free and the tumor-bearing areas were captured in each section and were analyzed using color deconvolution and Image-based Tool for Counting Nuclei (ITCN) plug-ins of ImageJ v1.45 software (National Institutes of Health, Bethesda, MD) by two observers blinded to treatment groups as per a protocol described earlier [33], [34]. Mean data from 6 mice are presented graphically as average arbitrary pixel unit of diaminobenzidine color intensity per 20X field (for β-catenin) or as average number of positive nuclei per 20X field (for p-H3, nuclear β-catenin, and cyclin D1) and a representative image (20X magnification) from one animal of each group is shown in the results. Data in figures are presented as mean ± standard error of mean (SEM).

### Immunoblots

For immunoblots, frozen tumor samples from 5 mice in each group were pooled and homogenized in ice-cold lysis buffer (0.5% sodium deoxycholate; 0.5% NP-40; 10mM EDTA in PBS) containing protease inhibitor cocktail (Sigma, St. Louis, MO). Lysate was centrifuged at 12000xg at 4°C for 15 m, and supernatant was used for protein estimation using a Bradford protein assay kit (Bio-Rad, Hercules, CA). Supernatant containing equal amounts of protein was further mixed with appropriate volume of 6X Lammeli gel loading buffer and incubated at 100°C for 5 min, followed by a brief centrifugation. Proteins were separated by sodium dodecyl sulphate-polyacrylamide gel electrophoresis (SDS_PAGE) and transferred onto polyvinylidene fluoride (PVDF) membrane, blocked with 5% non-fat milk (Bio-Rad) in tris-buffered saline with 0.1% Tween (TBST), and incubated with appropriate primary antibody β-catenin (Cell Signaling Technology, Danvers, MA; Cat#9562; dilution: 1∶500), phospho-β-catenin (Cell-Signaling Technology; Cat#9461; dilution: 1∶200), and phospho-GSK3β Ser-9 (Cell-Signaling Tech, Cat#9336S, dilution: 1∶200). Horseradish peroxidase (HRP) conjugated secondary antibody and enhanced chemiluminescence (ECL) detection system (Cat# 34080, Thermo Fisher Scientific, Rockford, IL) were used for developing the immunoblots. Images were captured on photographic films, scanned, and representative results are displayed. Densitometric quantification of immunoblot images was performed using ImageJ v1.45 software.

### Statistical Analysis

Data in figures are presented as mean ± standard error of mean (SEM). Differences between the groups were analyzed using two-tailed paired student’s *t* test. Results were considered significant at *p*<0.05.

## Results

### Heavy ion ^56^Fe Radiation Exposure Induced Higher Intestinal Tumorigenesis than γ Radiation in APC^Min/+^ Mice

**Figure 1:**
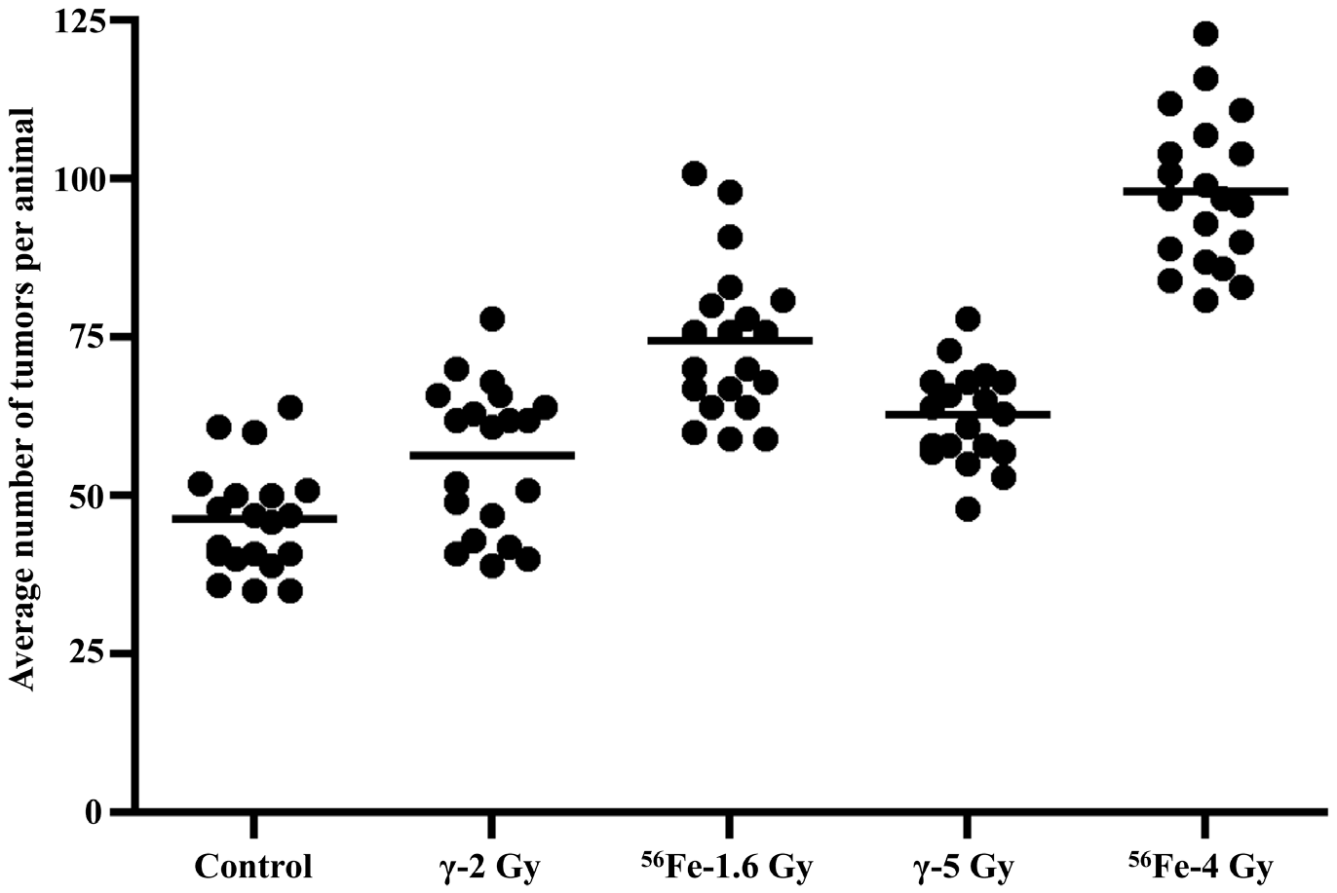
Heavy ion radiation-induced intestinal tumorigenesis in APC^Min/+^ mice. Intestinal tumor induction after 1.6 Gy or 4 Gy of ^56^Fe radiation is compared to respective equitoxic doses γ radiation (2 Gy γ equitoxic to 1.6 Gy ^56^Fe and 5 Gy γ equitoxic to 4 Gy ^56^Fe) as well as to sham-irradiated controls.

Exposure to ^56^Fe radiation caused markedly increased intestinal tumorigenesis relative to γ radiation in APC^Min/+^ mice (n = 20 per study group). While average number of tumors in the unexposed control group was 46.3±7.7 SD (standard deviation), the number of tumors in 2 and 5 Gy γ-irradiated mice was 56.3±11.6 SD and 62.7±6.8 SD respectively (**Figure 1**). Compared to control groups tumorigenesis was significantly higher after 5 (p<0.02) but not after 2 (p>0.22) Gy γ irradiation. Also, tumorigenesis after 5 Gy was not significantly different than the results obtained after 2 Gy γ radiation (p>0.09). Tumorigenesis after 1.6 (average tumor: 74.4±12.1 SD; p<0.004 compared to control) and 4 Gy (average tumor: 98±11.9 SD; p<0.0002 compared to control) ^56^Fe radiation was significantly higher relative to control (**Figure 1**). In contrast to γ irradiation, we observed a significant difference in tumorigenesis between 1.6 and 4 Gy ^56^Fe irradiation (p<0.01 compared between 1.6 and 4 Gy ^56^Fe). When tumorigenesis was compared between two radiation types, we observed significantly higher tumor numbers in ^56^Fe-irradiated groups relative to corresponding equitoxic dose of γ radiation (p<0.02 comparing 2 Gy γ and 1.6 Gy ^56^Fe; p<0.002 comparing 5 Gy γ and 4 Gy ^56^Fe; **Figure 1**).

**Figure 2:**
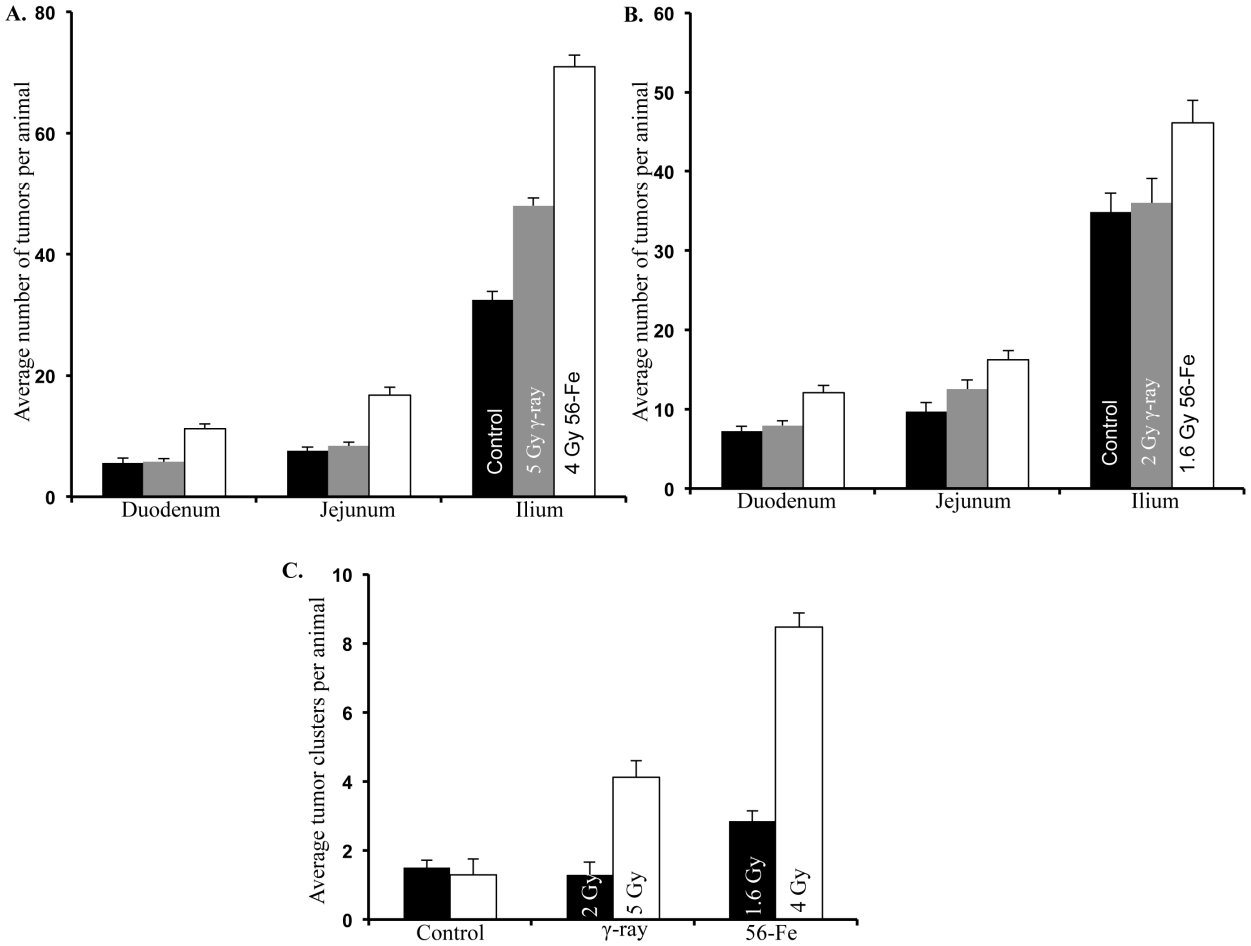
Tumor clusters and segmental distribution of tumors in intestine. A) Distribution of small intestinal tumors in duodenum, jejunum, and ilium after exposure to 5 Gy γ and equitoxic 4 Gy ^56^Fe. B) Distribution of small intestinal tumors in duodenum, jejunum, and ilium after exposure to 2 Gy γ and equitoxic 1.6 Gy ^56^Fe. C) Occurrence of tumor clusters defined as 5 or more tumors without intervening normal tissue was dependent on radiation quality and clusters were more after both 1.6 and 4 Gy ^56^Fe relative to respective equitoxic γ radiation doses.

The segmental distribution showed more tumors in the ilium than in the duodenum and jejunum, and tumorigenesis was higher after ^56^Fe radiation relative to γ radiation. Tumor numbers in ilium both at the lower doses (2 Gy γ: 36±13 SD; 1.6 Gy ^56^Fe: 46±13 SD; p<0.04) and at the higher doses (5 Gy γ: 48±6 SD; 4 Gy ^56^Fe: 71±9 SD; p<0.02) were significantly more after ^56^Fe radiation relative to γ radiation (**Figure 2A and B**). Tumor numbers in duodenum (for lower doses −2 Gy γ: 8±2 SD; 1.6 Gy ^56^Fe: 12±4 SD; p<0.01; for higher doses −5 Gy γ: 6±3 SD; 4 Gy ^56^Fe: 11±4 SD; p<0.02) and jejunum (for lower doses −2 Gy γ: 13±5 SD; 1.6 Gy ^56^Fe: 16±5 SD; p<0.04; for higher doses −5 Gy γ: 8±3 SD; 4 Gy ^56^Fe: 17±6 SD; p<0.007) were significantly higher after both the doses of ^56^Fe relative to γ radiation (**Figure 2A and B**). Tumor clusters, defined as 5 or more tumors without intervening tumor free areas, was significantly higher after ^56^Fe relative to γ radiation (**Figure 2C**). While it was similar in control and 2 Gy γ radiation (average cluster 1.5±1 in control and 1.3±1 in γ radiation, p>0.6), the number of tumor clusters was ∼2-fold higher in 1.6 Gy ^56^Fe-irradiated mice (average cluster 2.85±1.6 SD, p<0.01 relative control and p<0.003 relative to γ radiation). Tumor clusters were also significantly higher in 5 Gy γ relative to control (5 Gy γ: 4.12±2.27; control: 1.3±1.41, p<0.01). Notably, 4 Gy ^56^Fe radiation caused >2-fold increase in tumor cluster relative to 5 Gy γ radiation (average cluster 8.48±2.27, p<0.009 relative to control and p<0.0007 relative to γ radiation).

### Exposure to ^56^Fe Radiation Led to Increased Incidence of Larger and Higher-grade Intestinal Tumors

**Figure 3:**
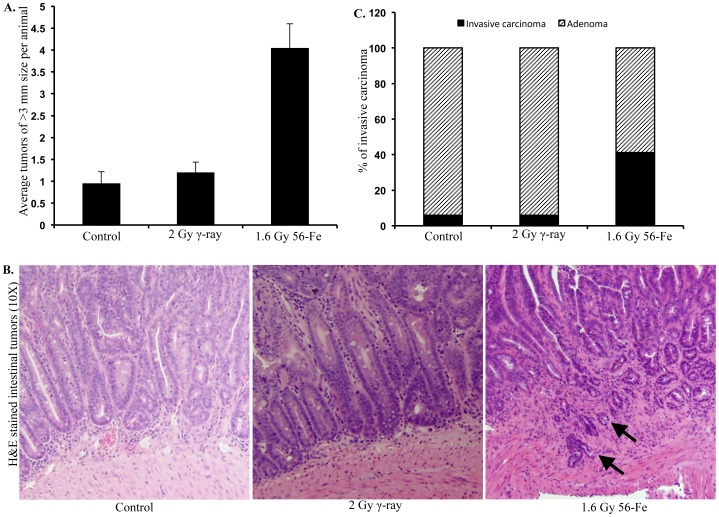
^56^Fe radiation-induced larger and higher-grade intestinal tumors. A) Compared to γ radiation, exposure to ^56^Fe radiation led to greater number of tumors whose size was ≥3 mm. B) H&E stained intestinal tumors showing crypt penetration of mucularis mucosa (arrow) indicating invasive adenocarcinoma after ^56^Fe radiation. Tumors in control and γ irradiated mice were mostly adenomas. C) Percent of invasive adenocarcinoma in control, γ, and ^56^Fe irradiated tumors.

When intestinal tumors after 2 Gy γ and equitoxic 1.6 Gy ^56^Fe radiation were categorized into two groups depending on their size (group 1: tumor size <3 mm and group 2: tumor size ≥3 mm), we observed that there were significantly higher numbers of larger tumors (tumor size ≥3 mm: average 4 tumors per animal; p<0.00001 compared to control and p<0.0001 compared to γ radiation) in ^56^Fe-irradiated mice relative to control and γ irradiated groups (tumor size ≥3 mm: average 1 tumors per animal; **Figure 3A**). Number of larger tumors after 5 Gy γ radiation was similar to 2 Gy. After 4 Gy ^56^Fe radiation there was, relative to 1.6 Gy ^56^Fe radiation, a small increase in larger tumor number which was not statistically significant (data not shown). Histopathological analysis of the intestinal tumors were performed in lower doses for their greater relevance to clinical and space travel scenario than higher doses and showed that 41.1% of the tumors after exposure to 1.6 Gy ^56^Fe radiation was invasive adenocarcinoma. In contrast only 5.8% of the intestinal tumors in the control and 2 Gy γ irradiated groups were invasive adenocarcinoma. Representative images of the tumors and percent of adenoma vs. invasive adenocarcinoma are shown in **Figure 3B and C**.

### Reduced Differentiation and Increased Proliferation of IEC after ^56^Fe Radiation

**Figure 4:**
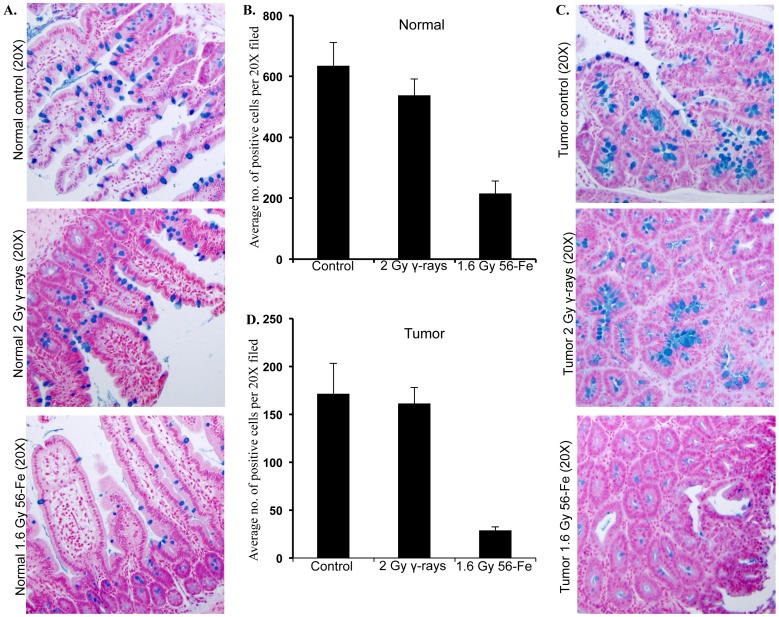
Alcian blue staining of intestinal sections showed reduced goblet cells after ^56^Fe radiation. A) Representative images of alcian blue stained tumor free (normal) areas of intestinal sections from control, γ, and ^56^Fe-irradiated mice. B) Quantification of alcian blue positive cells in tumor free areas of control, γ, and ^56^Fe-irradiated sections. C) Representative images of alcian blue stained tumor bearing areas of intestinal sections from control, γ, and ^56^Fe-irradiated mice. D) Quantification of alcian blue positive cells in tumor bearing areas of control, γ, and ^56^Fe-irradiated sections.

**Figure 5:**
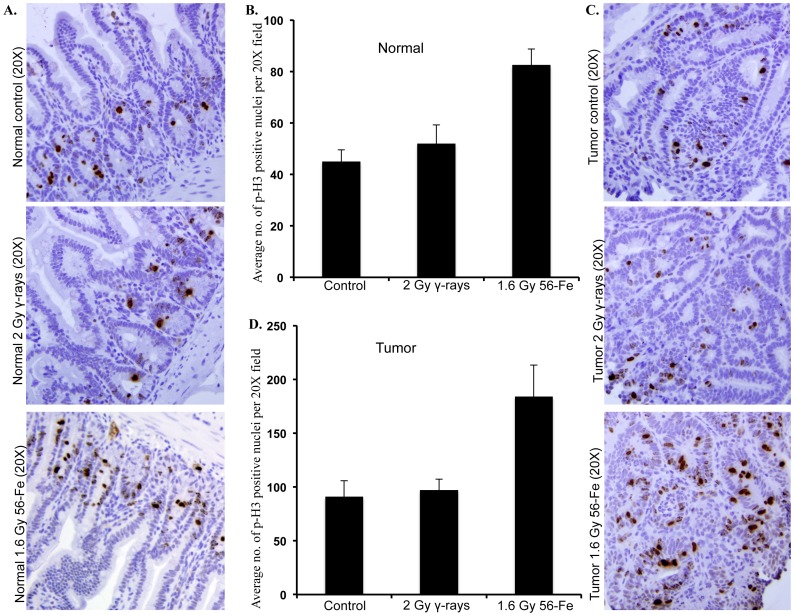
Greater phospho-histone H3 staining after ^56^Fe radiation. A) Representative images of phospho-histone H3 stained tumor free (normal) areas of intestinal sections from control, γ, and ^56^Fe-irradiated mice. B) Quantification of phospho-histone H3 positive nuclei in tumor free areas of control, γ, and ^56^Fe-irradiated sections. C) Representative images of phospho-histone H3 stained tumor bearing areas of intestinal sections from control, γ, and ^56^Fe-irradiated mice. D) Quantification of phospho-histone H3 positive nuclei in tumor bearing areas of control, γ, and ^56^Fe-irradiated sections.

Mucus-secreting goblet cells that can be stained by alcian blue have long been considered a marker of IEC differentiation and its rhythmic self-renewal process [35]. Relative to control and γ radiation, exposure to ^56^Fe radiation led to marked decrease in goblet cell numbers in both tumor-free (normal) and tumor-bearing areas of intestinal sections (**Figure 4A and C**). Quantification showed significantly greater decreases, relative to control and γ radiation, of alcian blue positive cells after ^56^Fe radiation in tumor-free (p<0.001 compared to control and p<0.004 compared to γ radiation) as well as in tumor-bearing (p<0.0002 compared to control and p<0.0008 compared to γ radiation) areas (**Figure 4B and D**). Staining of intestinal sections for phospho-histone H3 (p-H3), an indicator of cell proliferation [36], showed higher number of positively stained nuclei in tumor-free as well as in tumor-bearing areas of ^56^Fe-irradiated samples relative to control and γ radiation (**Figure 5A and C**). Counting showed significantly more positively stained nuclei in tumor-free (p<0.008 compared to control and p<0.02 compared to γ radiation) and tumor-bearing (p<0.01 compared to control and p<0.03 compared to γ radiation) areas of ^56^Fe-irradiated mice relative to control and γ radiation (**Figure 5B and D**). Staining in γ irradiated sections was not significantly different than sham-irradiated controls (p>0.76).

### Heavy Ion Radiation Induced Greater Activation of β-catenin

**Figure 6:**
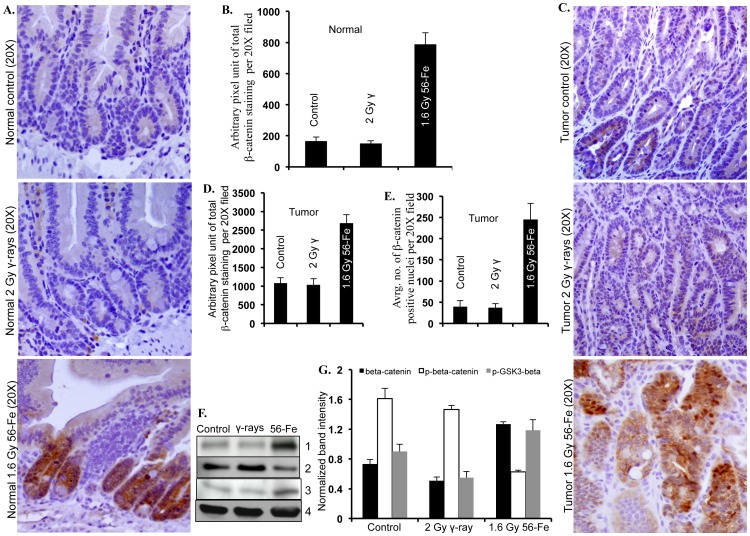
Increased activation of β-catenin after ^56^Fe radiation. A) Representative images of β-catenin stained tumor free (normal) areas of intestinal sections from control, γ, and ^56^Fe irradiated mice. B) Quantification of total β-catenin staining in tumor free areas of control, γ, and ^56^Fe irradiated sections. C) Representative images of β-catenin stained tumor-bearing areas of intestinal sections from control, γ, and ^56^Fe irradiated mice. D) Quantification of total β-catenin staining in tumor-bearing areas of control, γ, and ^56^Fe irradiated sections. E) Quantification of nuclear β-catenin staining in tumor-bearing areas of control, γ, and ^56^Fe irradiated sections. F) Immunoblots showing increased β-catenin, decreased phospho-β-catenin, and increased phospho-GSK3β after ^56^Fe radiation. Panel 1: β-catenin, 2: phospho-β-catenin, 3: phospho-GSK3β, 4: β-actin G) Quantification of immunoblots, normalized to β-actin band intensity, showed greater increase of β-catenin, decrease of phospho-β-catenin, and increase of phospho-GSK3β after ^56^Fe radiation relative to control and γ radiation.

**Figure 7:**
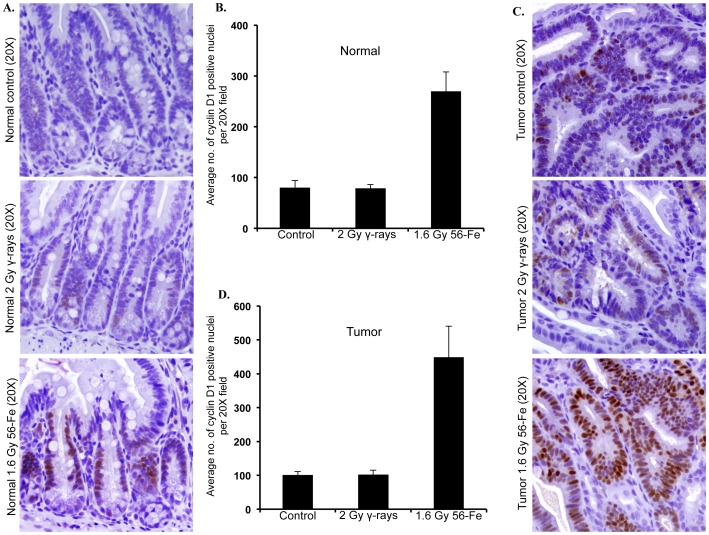
Cyclin D1 staining is greater after ^56^Fe radiation. A) Representative images of cyclin D1 stained tumor free (normal) areas of intestinal sections from control, γ, and ^56^Fe-irradiated mice. B) Quantification of cyclin D1 staining in tumor free areas of control, γ, and ^56^Fe-irradiated sections. C) Representative images of cyclin D1 stained tumor bearing areas of intestinal sections from control, γ-, and ^56^Fe-irradiated mice. D) Quantification of cyclin D1 staining in tumor bearing areas of control, γ, and ^56^Fe-irradiated sections.

Immunohistochemistry for active β-catenin showed markedly increased staining in both the tumor-free and tumor-bearing areas of intestine from ^56^Fe-irradiated mice (**Figure 6A and C**). Quantification showed significantly higher staining in both the tumor-free and tumor-bearing areas in ^56^Fe-irradiated mice relative to control and γ radiation groups (**Figure 6B, D, and E**). Importantly, both total and nuclear β-catenin staining in tumor-free (**Figure 6B**; p<0.004 compared to control and p<0.006 compared to γ radiation, and data not shown) as well as in tumor-bearing (**Figure 6D**; p<0.007 compared to control and p<005 compared to γ radiation, and **Figure 6E**; p<0.0002 compared to control and γ radiation) areas were significantly higher after 1.6 Gy ^56^Fe radiation relative to control and 2 Gy γ radiation. Beta-catenin staining after both 2 and 5 Gy γ radiation was not significantly different from the control groups (**Figure 6D** and **Figure S1**). Immunoblot analysis of tumor samples showed increased levels of β-catenin accompanied by decreased phospho-β-catenin and increased phospho-GSK3β after ^56^Fe radiation compared to control and γ radiation groups (**Figure 6F**). Quantification of band intensity normalized to β-actin showed significantly higher levels of β-catenin (p<0.006 compared to control and p<0.004 compared to γ radiation), and reduced phospho-β-catenin (p<0.0002 compared to control and p<0.0007 compared to γ radiation) (**Figure 6G**). We also observed increased phospho-GSK3β (p<0.008 compared to control and p<0.005 compared to γ radiation), indicating inactivation and thus inability of the protein to phosphorylate β-catenin, in ^56^Fe-irradiated tumor samples relative to control and γ radiation (**Figure 6G**). Cyclin D1 staining in tumor-free as well as tumor-bearing areas was higher after ^56^Fe radiation relative to control and γ radiation (**Figure 7A and C**). Quantification and statistical analysis showed, compared to control and γ radiation, significantly higher levels of cyclin D1 in tumor-free (p<0.0004 compared to control and γ radiation) as well as in tumor-bearing (p<00006 compared to control and γ radiation) areas after ^56^Fe radiation (**Figure 7B and D**).

## Discussion

A major concern for astronauts undertaking long duration space missions is exposure to heavy ion radiation such as ^56^Fe. Heavy ion radiation due to their high-LET characteristics and unique energy deposition pattern, which is highly heterogeneous, deposits higher amount of energy/unit area and also produces spectrum of energetic secondary nuclei [10], [37] in the traversed cells leading ultimately to a higher transformation potential. Indeed, higher carcinogenic potential of high-LET radiation has been reported in rodents and most of these initial studies involved neutrons [38]–[40]. High-LET-radiation-induced solid tumors were studied in tissues such as lung, mammary gland, ovary, and hardarian glands [38]–[41]. However, intestinal tumorigenesis and alterations in molecular pathways relevant to CRC after heavy ion ^56^Fe radiation exposure has not been studied in APC^Min/+^ mice. We show for the first time that ^56^Fe radiation-induced intestinal tumorigenesis was significantly higher at both the doses (1.6 and 4 Gy ^56^Fe) relative to corresponding eqitoxic γ radiation doses (2 and 5 Gy γ). Radiation therapy of human cancer commonly employs 2 Gy daily fraction of γ radiation to achieve the planned total dose [42]. Additionally, because long duration space mission is expected to expose astronauts to a cumulative heavy ion radiation dose of ≤1 Gy [6] and because the heavy ion radiation dose of 1.6 Gy is the lowest relevant dose in this study, we performed histopathological and molecular marker analysis relevant to CRC in 2 Gy γ and equitoxic 1.6 Gy ^56^Fe radiation. We demonstrate that 1.6 Gy ^56^Fe-induced intestinal tumorigenesis was associated with reduced IEC differentiation, increased cellular proliferation, higher tumor grade, and greater activation of β-catenin both in the tumors per se as well as in the normal-appearing mucosa relative to 2 Gy γ radiation. The RBE for the relative increase in intestinal tumorigenesis in our study was calculated to 2.8 and 3.05 for 1.6 and 4 Gy ^56^Fe respectively in relation to tumor incidence in γ-irradiated mice when the increases over the frequency in unirradiated mice were taken into account. This is higher than the RBE of survival (1.25) [27] and supports the notion that RBE for long-term effects of high-LET radiation such as cancer is higher than the short-term effects such as survival [38].

Previous studies have shown that high-LET particle radiation has greater effectiveness than low-LET radiation in inducing solid tumors and that tumorigenesis is dependent on a number of factors including total radiation dose received [38]–[41]. While tumors in excess of control mice in our study after 4 Gy ^56^Fe was more (average tumor: 52 per animal) than 1.6 Gy (average tumor: 28 per animal), the ratio of tumor increase from 28 to 52 was less (52/28 = 1.8) relative to the ratio of radiation dose increase from 1.6 to 4 Gy (4/1.6 = 2.5) suggesting proportionately greater increase after the lower dose. Indeed, tumor increase per unit of ^56^Fe radiation (cGy) showed greater increase after the lower dose than the higher dose (28/160 = 0.18 tumors per cGy after 1.6 Gy compared to 52/400 = 0.13 per cGy after 4 Gy). While tumorigenesis per unit of γ radiation (cGy) showed similar trend (10/200 = 0.05 tumors per cGy after 2 Gy compared to 16/500 = 0.03 per cGy after 5 Gy) as the ^56^Fe radiation, the number of tumors per cGy of γ radiation was markedly less than equitoxic ^56^Fe radiation (0.05 vs. 0.18 tumors per cGy after 2 Gy γ and 1.6 Gy ^56^Fe radiation respectively and 0.03 vs. 0.13 tumors per cGy after 5 Gy γ and 4 Gy ^56^Fe radiation respectively). Taken together our results does point to greater effectiveness of a relatively low dose of ^56^Fe radiation in intestinal tumor induction than the equitoxic γ radiation dose. We believe that in addition to physical differences in energy deposition between the two types of radiation, higher tumorigenic potential of heavy ion ^56^Fe radiation is due to higher transformation potential via differential gene-mutations [43]–[45], distinct gene expression/micro-RNA profile [46]–[48], and discrete damage response pathways and oncogenic signaling compared to low-LET radiation [49].

Studies have demonstrated that high-LET radiation carcinogenesis at lower doses increases linearly with radiation dose but at higher doses the response gradually plateaus which further led us to rationalize characterization of molecular events at lower doses. Additionally, International Commission on Radiological Protection (ICRP) has concluded that even for low-LET radiation there is increased risk of cancer at lower doses and proposed a linear no-threshold relationship [50]. Much of the oncogenic signaling events involved in colorectal carcinogenesis have been revealed through *in vitro* and *in vivo* studies [51]–[53] and activation of β-catenin has been recognized as a key event in the initiation and progression of CRC [54], [55]. Our immunohistochemistry and immunoblot data after 1.6 Gy ^56^Fe radiation showing increased β-catenin and reduced phospho-β-catenin indicates greater availability of active β-catenin relative to equitoxic 2 Gy γ radiation. Assessment of β-catenin immunohistochemistry staining after 4 Gy ^56^Fe radiation also showed similar trend as has been observed with the lower dose and the level was much higher relative to control and 5 Gy γ radiation. However, our results do indicate to a possible saturation effect both for the tumorigenesis (tumor numbers at 1.6 Gy is already 75% of the 4 Gy tumors) as well as for the β-catenin staining (β-catenin staining level at 1.6 Gy (2687 pixel unit) is already 88% of the 4 Gy staining (3049 pixel unit)) at higher doses of ^56^Fe radiation. Interestingly, although we observed a small increase, the β-catenin staining after 5 Gy γ radiation was not significantly different from the control group. Although both tumorigenesis and β-catenin staining are much less than ^56^Fe radiation, we again suspect that a saturation effect is in play for both the tumorigenesis (tumor numbers at 2 Gy is already 88% of the 5 Gy tumors) and the β-catenin staining (β-catenin staining level at 2 Gy (1035 pixel unit) is already 75% of the 5 Gy staining (1370 pixel unit)) at higher doses of γ radiation. It is believed that saturation effects of radiation-induced tumorigenesis is due to ineffective multiple traversal of a cell by radiation tracks as well as due to depletion target cells (potential tumorigenic cells) as a result of apoptosis at higher doses rather than survive and proliferate with damage, which is anticipated at lower radiation doses [56]. One of the mechanisms of increased β-catenin activity in APC^Min/+^ mice with germ line mutation in one APC allele is due to loss of the remaining wild type allele leading to loss of heterozygocity (LOH) [57], [58]. Wide variation in the frequency of LOH of APC gene (31 to 70%) has been observed for human CRC and APC^Min/+^ mice exposed to whole body 2 Gy x-ray radiation showed loss of the wild type allele (LOH) in about half of the intestinal tumors [59], [60]. Consequently, other somatic events in APC gene such as intragenic mutations, interstitial deletion, mitotic recombination, and epigenetic silencing of the wild type allele resulting in APC function loss has been proposed as additional mechanisms of intestinal tumorigenesis [57], [58]. Furthermore, reports also suggest that the truncated protein from the mutated allele may exert a dominant negative effect on the full-length protein from the wild type allele of the APC gene resulting in a non-functional APC protein [16]. Currently, efforts are underway in our laboratory to understand frequency and characteristics of ^56^Fe radiation-induced LOH at the APC locus on mouse chromosome 18 in F1 AKRxC57BL/6 J-Apc^Min/+^ hybrid mice, commonly used for such studies [60]. Considering that β-catenin and its downstream effector cyclin D1 was increased in the tumor-free as well as in the tumor-bearing areas of the intestine after ^56^Fe radiation, we speculate that one or more of the additional APC-independent mechanisms of β-catenin stabilization discussed above could be involved in ^56^Fe radiation-induced intestinal tumorigenesis. Furthermore, the fact that about half of the x-ray induced intestinal adenomas in APC^Min/+^ mice arise without LOH and most of the adenomas are polyclonal supports the notion that not all the intestinal tumors results from functional APC loss mediated β-catenin stabilization [57], [61], [62]. Indeed, stabilizing mutations of the β-catenin, loss of function mutation of scaffolding protein axin as well as gain of function alterations in transcription factor TCF4 have all been implicated in cellular accumulation and increased activity of β-catenin leading to intestinal tumorigenesis [62]. However, we are yet to accumulate evidence to either support or refute roles of these additional mechanisms in ^56^Fe-induced enhanced intestinal tumorigenesis.

Scaffolding protein axin along with APC is essential for GSK3β mediated β-catenin phosphorylation and activity of GSK3β, a constitutively active protein kinase, is inhibited by phosphorylation [63]. Indeed, inactivating phosphorylation of GSK3β by upstream kinases such as Akt (protein kinase B), protein kinase C (PKC), and cyclic AMP dependent protein kinase (protein kinase A) has been shown to decrease phosphorylation and increase stability of β-catenin resulting in its cytoplasmic accumulation, and nuclear translocation [64], [65]. Therefore, our immunoblot results showing preferentially increased phospho-GSK3β in ^56^Fe-irradiated tumor samples suggest an additional route of β-catenin activation and intestinal tumorigenesis after space radiation. Nonetheless, increased accumulation of active β-catenin results in its subsequent nuclear translocation and in conjunction with TCF4 causes transcription of target genes such cyclin D1 leading to higher cellular proliferation [66]. Indeed, higher proliferation not only in tumor-free intestinal tissues but also in the tumor-bearing areas leading to greater tumorigenesis and more tumors per unit area was observed after ^56^Fe radiation exposure in APC^Min/+^ mice. Consequently, phosphorylation of histone H3 at ser-10, known to accompany transcriptional activation as well as cell division and proliferation [36], are more in ^56^Fe-irradiated mice. Importantly, ^56^Fe radiation exposure not only increased proliferation of IECs but it also reduced as evident from decreased goblet cell staining their differentiation. We propose that ^56^Fe radiation-induced increased proliferation and decreased differentiation of IEC is promoting not only larger but also higher-grade intestinal tumors. It has been suggested that a major basis of carcinogenesis is blockage in cellular differentiation resulting in maintenance of stemness in incompletely differentiated cells [67]. We believe that incompletely differentiated IECs with their stemness intact under the influence of increased β-catenin proliferative signals are driving higher tumorigenesis in ^56^Fe-irradiated mice. However, detail investigation will be required to determine the mechanisms of how space radiation differentially affects intestinal stem cell, and its niche and onocogenic signaling, which we believe are playing important roles in ^56^Fe radiation induced higher intestinal tumorigenesis. Taken together our current results in APC^Min/+^ mice suggest that there is an increased risk of CRC due to greater activation of β-catenin pathway after heavy ion ^56^Fe radiation exposure.

### Conclusions

Knowledge of long-term effects after exposure to heavy ion radiation have implications for health risk estimates of not only astronauts undertaking exploratory missions into outer space and future space tourists but also of patients exposed to heavy ion radiotherapy. We have demonstrated that relative to control and γ irradiation, ^56^Fe radiation induced more tumors not only after 4 Gy but also after 1.6 Gy. We further demonstrate that compared to γ radiation, exposure to 1.6 Gy ^56^Fe radiation was associated with lower differentiation and higher proliferation of IEC, and higher grade intestinal tumors. Exposure to heavy ion radiation was also associated with higher β-catenin and cyclin-D1 levels than γ radiation. Taken together our results show that heavy ion ^56^Fe radiation induced differential upregulation of β-catenin and its downstream effectors, which we believe is playing a role in higher intestinal tumor number and grade after ^56^Fe radiation exposure in APC^Min/+^ mice. Finally, accumulation of *in vivo* mechanistic data on heavy ion radiation effects will not only allow us to estimate risks to human health but will also allow us to devise strategies to minimize risk from such radiation exposures.

## Supporting Information

Figure S1 Increased activation of β-catenin after 4 Gy ^56^Fe radiation. Quantification of total β-catenin staining in intestinal section of control, γ, and ^56^Fe irradiated mice. β-catenin was markedly greater after 4 ^56^Fe radiation relative to control and 5 Gy γ radiation. Staining in 5 Gy γ irradiated samples was similar to control.
(TIF)
