# Supplementary figures and images for: Heavy Ion Radiation Exposure Triggered Higher Intestinal Tumor Frequency and Greater β-Catenin Activation than γ Radiation in APCMin/+ Mice
Source: PLoS One. 2013 Mar 21;8(3):e59295. doi: 10.1371/journal.pone.0059295 (PMC3605451; doi:10.1371/journal.pone.0059295)

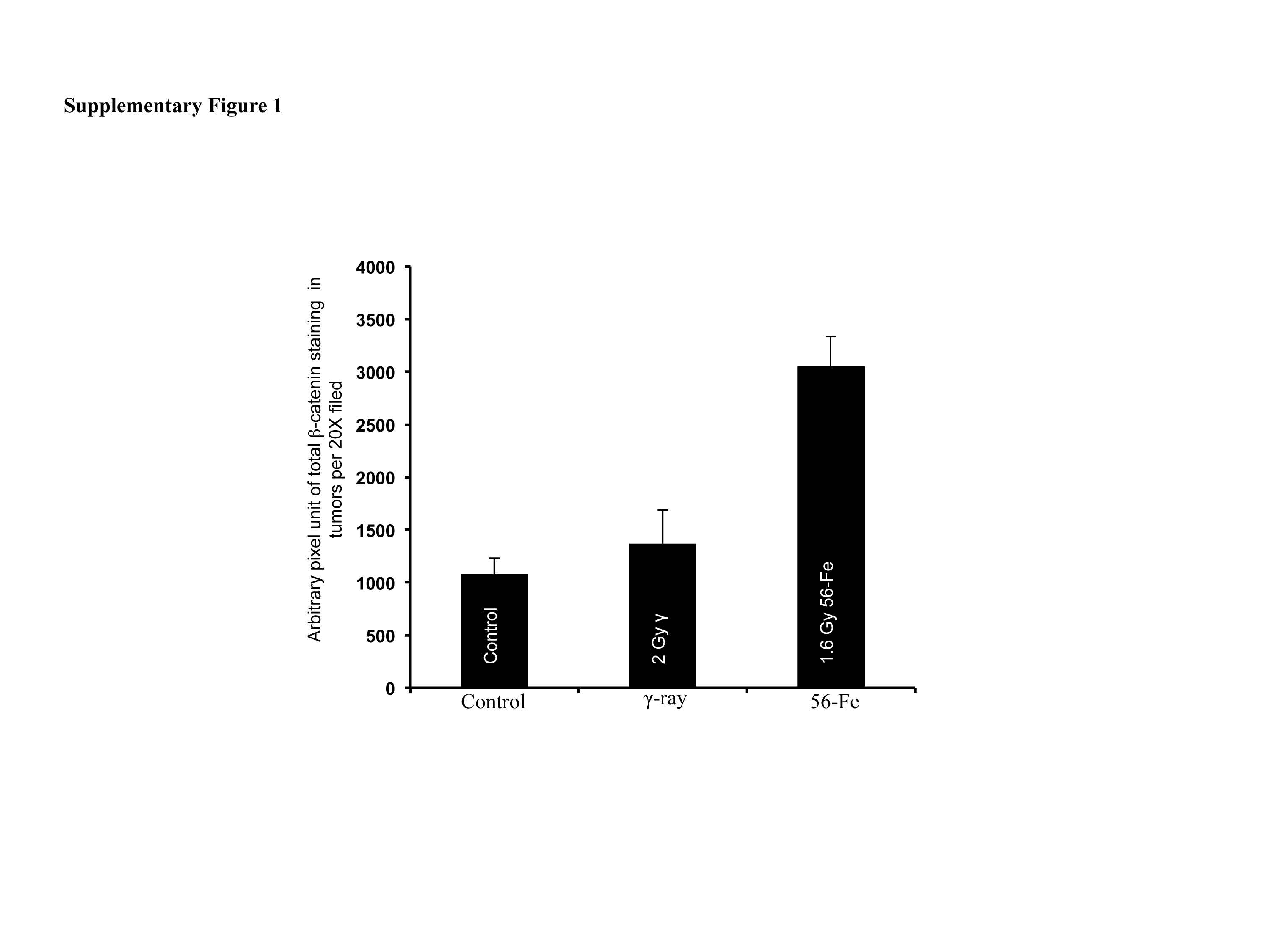

Supplement: Figure S1 — Increased activation of β-catenin after 4 Gy 56Fe radiation. Quantification of total β-catenin staining in intestinal section of control, γ, and 56Fe irradiated mice. β-catenin was markedly greater after 4 56Fe radiation relative to control and 5 Gy γ radiation. Staining in 5 Gy γ irradiated samples was similar to control. (TIF) [file pone.0059295.s001.tif]
